# Evaluation of Factors Affecting the Surgical Outcome in Tympanoplasty

**Published:** 2016-03

**Authors:** Masoud Naderpour, Yalda Jabbari Moghadam, Ensieh Ghanbarpour, Nikzad Shahidi

**Affiliations:** 1 *Department of Otorhinolaryngology, Tabriz University of Medical Science, East Azarbaijan, Iran.*

**Keywords:** Chronic suppurative otitis media, Graft, Tympanoplasty

## Abstract

**Introduction:**

Tympanoplasty is a standard procedure to repair tympanic membrane perforation. The aim of this study is to evaluate the results of tympanoplasty (hearing improvement and tympanic membrane closure rate) in patients suffering from chronic perforation of the tympanic membrane by considering the prognostic factors.

**Materials and Methods:**

In a prospective study, based on the results of tympanoplasty with temporal graft fascia in 60 patients in the ENT department of the Medical Science University of Tabriz, we evaluated prognostic factors, such as age, sex, smoking, size, and site of perforation, for the outcome of this surgery.

**Results:**

The rate of surgical success- integration of the graft- was 93.3%. Improvement of hearing, as demonstrated through audiometry, occurred in 93% of cases. We did not find any factors to be statistically signiﬁcant to affect surgical outcome.

**Conclusion:**

Even by considering the influence of different factors on the results of a tympanoplasty operation, according to the statistical results of this study, there is not a significant difference in the results of the operation, neither in the health of the tympanic membrane after surgery nor in hearing development.

## Introduction

Chronic otitis media (COM) is defined as an inflammation of the middle ear with signs of infection lasting three months or longer. Chronic suppurative otitis media (CSOM) is defined as a chronic inflammation of the middle ear and mastoid cavity, which presents with recurrent ear discharges or otorrhoea through a tympanic perforation. The presence of a persistent tympanic perforation and middle ear discharge differentiates CSOM from other forms of chronic otitis media (1). 

CSOM has been known since pre-historic time. Contributing factors are: low living conditions, poor personal hygiene, and diet (2). Perforation of the tympanic membrane (TM) can be caused by trauma and disease of the middle ear. Rupture of the membrane (perforation), which occurs as a result of chronic otitis media, involves at least 0.5% of the population. CSOM can cause conductive hearing loss up to 60 db, which is considered a serious disability (3). Tympanoplasty is a procedure that is used for COM treatment. It aims to rebuild the perforated ear drum and restore the function of the middle ear. Surgical approach for tympanoplasty can be endaural or transmeatal, postauricular, and superameatal. The most common technique of grafting is underlay (medial) and overlay (lateral). Temporalis muscle fascia and targal cartilage’s perichondrium are the most popular materials as a graft (4).

Many factors have been investigated to determine their effect on the tympanic membrane closure rate and hearing improvement. Various studies on tympanoplasty have been conducted, which show that success rate and criteria for success vary from author to author. Some studies demonstrated that surgical outcome depends on several factors including size and location of the perforation, ossicular status, type of surgical technique, graft type and function of the eustachian tube. However, according to many authors, surgical outcome is independent of factors that are deemed as relevant (3-10). 

It is difficult to compare these studies because of differences in age, definition of success, the method used, and the experience of the surgeon. Thus, the factors affecting tympanoplasty must be studied depend of the conditions of each study.

## Materials and Methods

A total of 60 patients who underwent tympanoplasty operations between June 2013 and September 2014 were reviewed in this study. The age of the patients was between 18 to 49 years. Underlying diseases such as diabetes or immunodeficiency, cholesteatoma and erosion ossicular, presence of sensorineural hearing impairment, definitive diagnosis of tympanosclerosis, revision cases were the exclusion criteria for the study. Patient evaluation was undergone through proper history noting and thorough examination. Examination was performed radiologically if needed. For example, if there was a suspected retraction pocket, the patient was evaluated with computer tomography. Examination could also be performed audiologically and finally examined under microscope. The operation method used on all patients was the same. The post auricular approach, underlay technique, and harvesting of the temporalis fascia as the graft material was used in all the tympanoplasty procedures and the surgeon and its assistant were stable in all surgeries. One week after the operation, the status of the surgical wound was evaluated and packing was removed. Patients were followed up postoperatively up to 3 months. During the follow-up, the condition of the wound and the tympanic membrane was noted. An audiogram was performed on the 12th weeks to assess the outcome. 

Data were analyzed using the chi-squared tes and t-test on a spss statistical package (version 16.0; SPSS). A P<0.05 was the level of significance.

## Results

For the 60 tympanoplasties, the male to female ratio was 1:2 and the age ranged between 18 to 49 years (mean 33.6 years; SD=7.32). The distribution of patients by age and sex is shown in Table.1.

**Table1:** Distribution of patients by age and sex

		**Sex**		**Total**
		**Male**	**Female**	
age	18-28	6	8	14
	29-39	10	20	30
	40-49	4	12	16
Total		20	40	60

The perforation sites were anterior maleolar (12 patients, 20%), posterior maleolar, (14 patients, 23.3%), and central (34 patients, 56.7%). The most common perforations were small (less than 50% of the tympanic membrane). 30% of patients were smokers and 70% were non-smoker.

**Fig 1:**
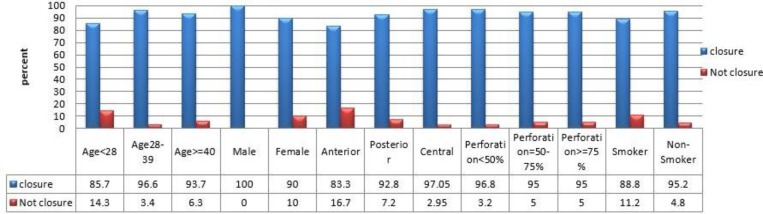
Tympanic membrane closure rates

The overall graft success rate was 93.3% (56 of 60 patients). Based on the univariate analysis, size of perforation (P=0.08), site of the perforation (P=0.26), smoking (P=0.36), age (P=0.36) and sex (P=0.143) no statistical signiﬁcance was observed as prognostic factors (Fig. 1).

The average air–bone gap improvement for all 60 tympanoplasty procedures was 18.8 dB±5.62 SD. Serviceable hearing (air–bone gap<20 dB) was achieved in 93.3% of the 60 tympanoplasties postoperatively. The mean preoperative air–bone gap was 28.41 dB±5.4 SD and the mean postoperative air–bone gap was 9.56 dB ± 6.07 SD. The inﬂuence of the prognostic factors on hearing improvement in the 60 tympanoplasties is shown in Table.2. No parameters were found to be statistically signiﬁcant for hearing improvement.

**Table 2:** Success rates in parameters reviewed

	**Total number**	**Success rate P-value**		**Post-op ABG** **≤ 20dB P-value**	
Age					
<28 years 28-39 ≥40 years	14 30 16	85.7% 96.6% 93.7%	0.37	92.8% 96.6% 87.5%	0.49
Sex					
male female	20 40	100% 90%	0.143	90% 95%	0.46
site of perforation					
Anterior Posterior Central	12 14 34	83.3% 92.8% 97.05%	o.26	83.3% 100% 94.1%	0.2
Size of perforation (%)					
‹50 50-75 ≥75	32 20 8	96.08% 95% 95%	0.08	93.5% 95% 88.8%	0.82
Smoking					
Smoker Non-smoker	18 42	88.8% 95.2%	0.36	94.4% 92.8%	0.82

## Discussion

Tympanoplasty is a surgical technique for the treatment of patients with chronic otitis media (COM). The goal of tympanoplasty is to eradicate disease and restore the function of the middle ear (11).

In order to repair tympanic membrane perforation, various materials including areolar connective tissue, fascia, prechonderium, periosteum, skin, fat, veins, and alloderm mucosa are used as a graft (5,11). In this study, we used a post auricular approach, underlay technique, and harvesting of the temporalis fascia as the graft material. In our study, tympanic membrane closure rates was 93.3%, which is similar to other studies (10,12-15) and even better than some studies (16,17). This difference in the levels of tympanic membrane closure rates may be due to different conditions of the middle ear throughout different studies.

The mean air-bone gap improvement in our study was 18.8 dB ± 5.62 SD and air-bone gap was significantly decreased postoperatively (P=0.002). Karela et al indicated hearing improvement in 91.5% of cases and stated that myrangoplasty is a procedure that can be successful in many cases, regardless of age, gender, location, and size of the perforation (6).

Some studies show age as a prognostic factor and stated that the success of the graft integration in children is slightly lower than in adults (9,18) and that this is due to the fact that children have persistent dysfunction of the Eustachian tube, recurrent infections of the respiratory tract with otorrhea, and lack of development of the immune system (10,19). In our study, the mean age was of 34.07±7.3 and the tympanic membrane closure rate and hearing improvement was similar to other studies; therefore, indicating age is not a prognostic factors (4,6,20).

In our study, females were predominant over males (60% vs 40%). However, there was no statistically significant correlation between sex and success rate, which was similar in other studies (1,6,21) .Emir and et al showed that being male was a good prognostic factor (9).

Based on several studies, the highest hearing loss occurs with the perforation of the large central and the lowest hearing loss occurs with the perforation of the anterior central. Larger perforations produce more pronounced losses; therefore, the size of perforation is an important factor for hearing loss. However, below 10% of the tympanic membrane, perforation size does not influence hearing (7,22). In our study, the highest hearing loss occurred with the perforation of the large anterior perforation and the lowest hearing loss occurred with a small posterior perforation.

Zhang and colleagues demonstrated that after myrangoplasty for small perforation of the tympanic membrane (less than 50%) ABG is minimum (average 5.5dB) and most ABG (average 10.5 dB) was after closure of large perforations (more than 50%) (23). Lee and et al report that the recovery air threshold after myrangoplasty is directly associated with the preoperative size of the tympanic membrane (10).

Karela and et al, examined the outcome of myrangoplasty and hearing improvement and stated that the size and location of the perforation has no effect on the tympanic membrane closure rate and hearing improvement (6). Other studies have also demonstrated that the perforation size does not affect the surgical outcome (24-26); while some studies show that perforation size does affects the outcome (8,27). In our study, the size of the perforation had no effect on tympanic membrane closure rate and hearing improvement after surgery.

Yung et al.'s study concluded that a large central, central maleolar, and central posterior perforation show the most hearing loss and that posterior inferior perforations cause greater hearing loss than anterior inferior perforations (28). Ahmad and Ramani showed that the difference in hearing loss between anterior and posterior inferior perforations is only seen at low frequencies (29).

There are several studies examining the effects of the location of the perforation on the surgical outcome. Jurovitzki stated that an anterior perforation was less favorable than perforations seen in other locations (30). Gonzalez observed better success for perforations of the posterior and weaker success in perforation of the subtotal (31). Albera showed that risk of tympanic reperforation is more common in posterior perforations (32); while Onal reported that the success rate of the anterior, posterior, and central perforation showed no difference (8). In our study tympanic membrane closure rate and hearing improvement was not associated with location of perforation, similar to other studies (4,6).

Controversy exists in the results of tympanic membrane closure rate and hearing improvement in smoker and non-smoker patients. In a study of 132 patients, performed by Cantrell, success rate in non-smokers was 92.5% and 43.7% in smokers (33).

Becavarovski, in the study of 74 tympanoplasty surgeries, showed that delayed complications of surgery, such as severe atelectasis or delayed perforation after 6 months, was 20% in non-smokers and 60% in smokers (P<0.5) (34).

Onal et al reported tympanic membrane closure rate to be 78.7% and 47.4% in non-smokers and smokers, respectively (P=0.008) (8).

 Other studies have not shown that smoking is a prognostic factor for graft integration, but did demonstrated that it had negative effects on the long-term results of surgery (4).In our study, smoking was not seen as an effect on tympanic membrane closure rate and hearing improvement; however, we did not follow patients for a long time.

## Conclusion

Even though many different factors can influence the results of a tympanaplosty operation, according to the statistical results of the study, there is no significant difference in the outcome of the operation, neither in the health of the tympanic membrane after surgery nor in hearing development. However, more studies on more samples in various centers sould be conducted in order to make an acceptable conclusion.
